# A Conservative Esthetic Approach Using Enamel Recontouring and Composite Resin Restorations

**DOI:** 10.1155/2016/1254610

**Published:** 2016-10-12

**Authors:** Paula Mathias, Emily Vivianne Freitas da Silva, Thaiane Rodrigues Aguiar, Aline Silva Andrade, Juliana Azevedo

**Affiliations:** ^1^Department of Clinical Dentistry, School of Dentistry, Federal University of Bahia, Salvador, BA, Brazil; ^2^Department of Dental Materials and Prosthodontics, Aracatuba Dental School, Universidade Estadual Paulista, UNESP, Aracatuba, SP, Brazil; ^3^Department of Restorative Dentistry, Araraquara Dental School, Universidade Estadual Paulista, UNESP, Araraquara, SP, Brazil; ^4^Department of Clinical Dentistry, School of Dentistry, Bahiana School of Medicine and Public Health, Salvador, BA, Brazil

## Abstract

Conservative clinical solutions, predictable esthetic, and immediate outcomes are important concepts of restorative dentistry. The aim of this case study was to recognize the selective enamel removal as an interesting conservative alternative to achieve optimal esthetic results and discuss the clinical protocol. This clinical report described an alternative esthetic and conservative treatment to transform the long and sharp aspect of the maxillary canines with a slightly aggressive aspect into features of slightly curved teeth with delicate lines. An accurate diagnostic and esthetic analysis of the smile was initially performed. The selective enamel removal was performed, and direct composite restoration was strategically placed. Clinical assessment showed good esthetic outcomes, enabling a smile harmony with an immediate, simple, and lower-cost technique. Practitioners should be exposed to conservative approaches to create esthetic smiles based on the selective enamel removal technique combined with composite resin.

## 1. Introduction

The esthetic demand for a Perfect Smile has been growing fast year-after-year, as an important way to improve the patient acceptance with tooth appearance especially related to color changes and alignment [1–3]. Overall, patients are more confident after dental treatment, which positively affect their personal lives and professional relationships [4–6]. On the other hand, esthetic treatment is always accompanied by unique expectations with regard to the final outcomes.

Facial esthetics are guided by the harmony of soft tissue, gingival esthetics, microesthetics, and macroesthetics [7–9]. To improve the dental esthetic appearance, restorative approach can be combined with interdisciplinary treatment, such as periodontal and orthodontic treatment [3, 10]. However, these techniques require time due to the healing process and changes of the alveolar bone.

Although interdisciplinary evaluation and treatment are crucial in achieving optimum smile composition, understanding the patient's wishes is essential to the success of therapy [3, 10]. Therefore, to cater to the patient requirement for improved dental esthetic results in the shortest possible time, selective enamel recontouring and direct restorations seem to be a good alternative [2, 11–15].

Minimally invasive treatment can be performed using selective enamel recontouring since the removal of a slight amount of enamel is only superficial, without dentin exposure and hence no postoperative sensitivity [2, 16]. In addition, the main advantage of this treatment is restoring a more natural teeth appearance using a relatively simple, immediate, and low-cost manner [2]. Thus, the aim of this article was to describe a successful conservative technique when changes in the shape of teeth are required for optimal esthetic results.

## 2. Clinical Report

**Figure 1:**
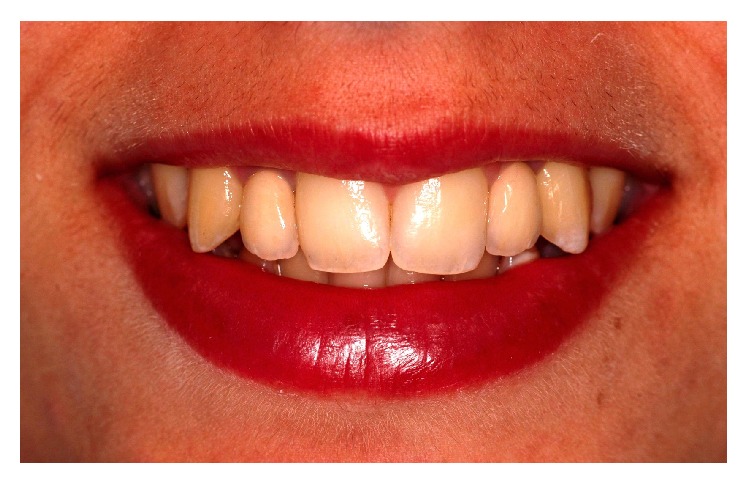
Initial clinical aspect showing the relationship between sharp maxillary canines and lips. Incisal thirds of the canines are projected in the vestibular direction, compromising tooth alignments and the harmony of smile.

**Figure 2:**
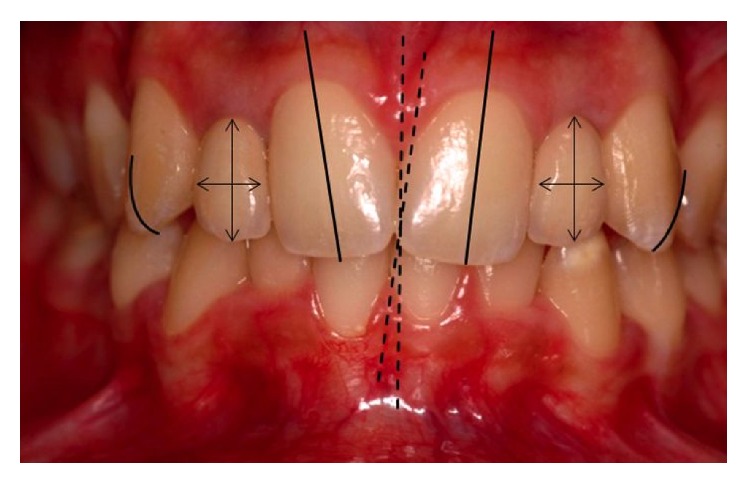
Frontal view showing the mid sagittal line alteration between the midline of the maxillary and mandibular arches (dotted lines); changes in the long axis of the maxillary central incisors (unbroken lines); disharmony in the height-width ratio of the lateral incisor crowns (crossed arrows); more prominent incisal thirds of the maxillary canines projected in the labial direction with sharp appearance (schematic drawing with curved lines pointing out the sharp aspect of canines).

A 26-year-old female patient complained about the elongated and protruded aspect of her maxillary canine (Figure 1). The patient agreed to treatment and signed an informed consent. An interview of her medical history was performed and no concern was revealed. After clinical examination and esthetic analysis, some alterations were noted: midline variation between the maxillary and mandibular dental arches; changes in the axial inclination of maxillary central incisors; disharmony between the height-width ratio of dental crowns on the lateral incisors; more pronounced proclined maxillary canines with a sharp aspect, giving her smile a more aggressive appearance and masculine characteristic (Figure 2).

Concerning the gingival esthetics, a cervical gingival gap in the region of the lateral incisors was observed in an intraoral photograph; however, it was not revealed in the smile photography. Therefore, since the patient did not complain about this aspect, no periodontal intervention for esthetic purpose was performed. In spite of recognizing the benefits of an orthodontic treatment, the patient reported satisfaction with the other aspects related to her smile and the color of her teeth. In addition, she was looking for immediate esthetic results and a minimally invasive manner. She also pointed out that she sought dental treatment at that time only to solve the esthetics of her smile as described before. It is emphasized out that the patient has dental implants and their respective implant supported dentures in the region of tooth #1.2 and tooth #2.2 (maxillary lateral incisors).

**Figure 3:**
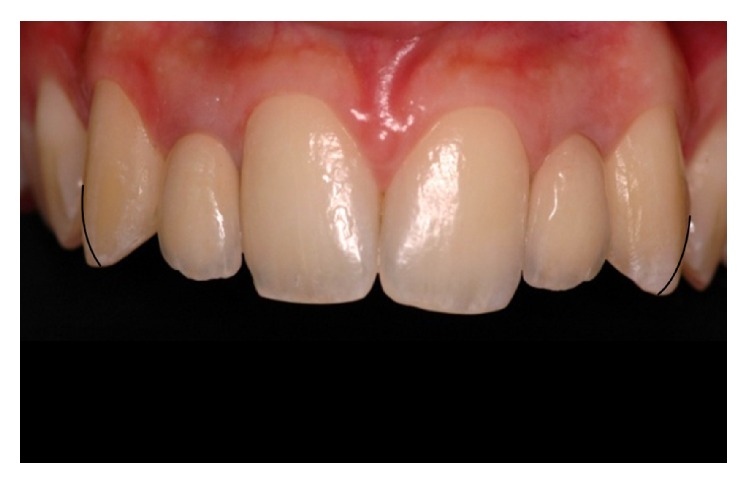
Frontal view of selective enamel recontouring of canine maxillary using a guide in order to reduce the buccal inclination, especially in the incisal third, and obtain an incisal angle more rounded.

**Figure 4:**
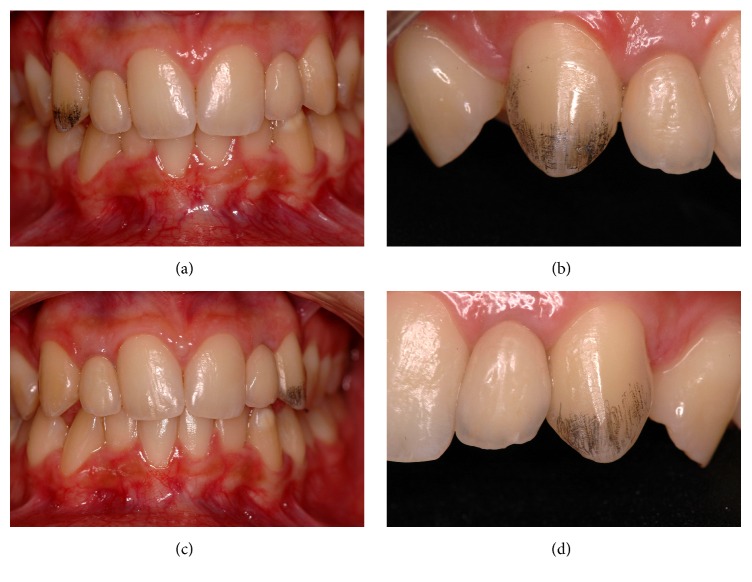
Different view of the guide of selective enamel recontouring of canine maxillary. (a) Marking on the most prominent third of the buccal face of tooth #13. (b) An approximate view of the teeth after enamel recontouring. (c) Marking on the most prominent third of the buccal face of tooth #23. (d) An approximate view of the teeth after enamel recontouring. Note on the proximal saliencies, greater wear was performed in the cervical direction in order to prevent them from remaining more projected and therefore outstanding in the smile.

Initially, occlusal contacts were verified with articulating paper. When the canine guidance was observed, the option taken in order to attend the patient's demand was not to perform any wear in the incisal and palatine surface. As a result, the selective enamel recontouring was planned without reduction of the length of the tooth. To control this procedure, the guide was done with graphite on each tooth (#1.3 and #2.3) (Figures 3 and 4). The enamel recontouring was performed with diamond tip (#9138, KG Sorensen, Cotia, SP, Brazil), at high speed under constant water irrigation. Afterwards, decreasing granulation of aluminum oxide discs (Sof-lex Pop-on, 3M ESPE, St. Paul, MN, USA) was used to refine and polish the enamel surface.

The reduction of the buccal inclination of the incisal third as well as the rounding of incisal edge towards the palatine surface was performed. As shown, the flat area of the buccal surface was reduced in the cervical-incisal direction, giving the teeth a less elongated appearance. In addition, the enamel recontouring promoted better alignment between the incisal third of maxillary canines and incisors. Moreover, the enhanced space in the buccal corridor makes the smile more attractive.

To give the canines a more rounded aspect, which characterizes a more feminine smile, composite resin restorations in the cervical to middle third of the buccal surface of the maxillary canines were planned [8]. Moreover, an additional small direct restoration was indicated at the distal incisal angle fractured of the permanent central incisor (#1.1). The composite resin selection was performed positioning small increments of the selected composite over the teeth, followed by its polymerization [17]. Afterwards, a relative isolation was provided using labial retractor, gingival retraction cord, and cotton rolls. Then, etching enamel with phosphoric acid 37% was performed followed by the application of the adhesive systems (Adper Scotchbond Multipurpose Primer/Adhesive, 3M ESPE). The resin composite (Supreme XT Shade A3-E, 3M ESPE) was applied in the cervical to middle third of the canines in order to reproduce the buccal enamel. For fractured incisor, the resin composite shades A2B and A2E (Supreme XT, 3M ESPE) were used to restore the edge and the external enamel, respectively. All materials were used in accordance with the manufacturer's instructions and light-activated using a light emitted diode unit (Bluephase, Ivoclar-Vivadent, Schaan, Liechtenstein).

**Figure 5:**
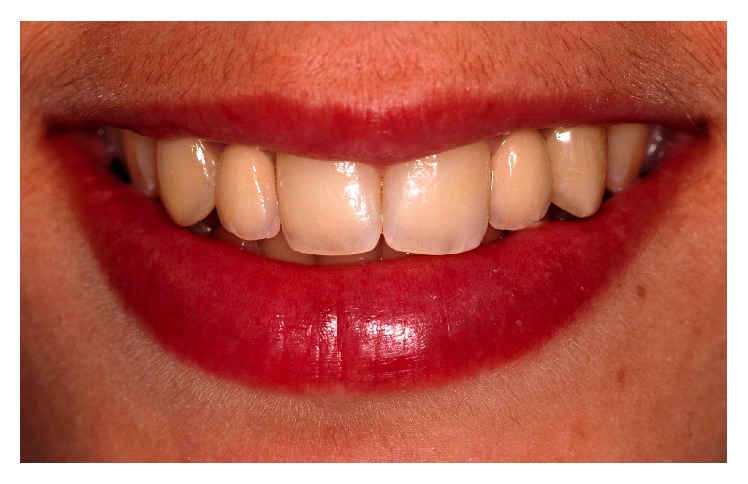
Final aspect after performing the finishing and polishing procedures, in which more harmonious and rounded contours, more in keeping with the patient's face and gender, may be observed.

Afterwards, the restorations were polished with decreasing grits of aluminum oxide discs. In the smile photograph, the obtainment of more harmonious shapes and sizes of maxillary canines can be observed (Figure 5).

## 3. Discussion

The improvement in self-esteem, quality of life, and social acceptance are important factors that induce the patient seeking esthetic dental treatment [4–6]. Optimizing smiles guided by preservation of tooth structure, immediate results, and eventual future replacements are a changeling in restorative dentistry field [18]. Although new techniques and restorative materials are constantly being developed, a conservative and reversible approach to improve the smile design can be obtained by implementing a relative simple technique based on selective enamel removal and composite resin restoration.

In this case presentation, we reported a predictable plan treatment to transform the sharp and aggressive aspect of maxillary canines into more rounded and harmonious design according to the patient's face and gender. Regarding the dental esthetic principles, tooth size, shape, and proportion between height and width of crowns are the most easy perceptible factors in the smile [7]. Moreover, dental morphology should be harmonious with the gender characteristics to make a more natural smile [8, 19]. The feminine tooth shows characteristics such as slightly curved, smooth, delicate lines and absence of sharp angles. In contrast, masculine teeth are more angular and have straight lines, giving them an aspect of force and greater aggressiveness [8].

Reshaping performed with restorative techniques such as selective enamel recontouring or additional composite resin has shown advantages. For instance, allow greater preservation of healthy tooth structure, few clinical sessions, and reduced cost when compared to indirect restorations [3, 11, 12, 15, 20, 21]. It is emphasized that, in spite of being a simple technique, it depends on adequate treatment planning including knowledge of tooth morphology and dental/dentofacial esthetic common sense principles [22, 23].

After treatment, canine crowns showed a more adequate volume and depth. These morphology characteristics improve the smile attractiveness [8]. Other studies have shown that enamel recontouring is a safe and widely used procedure for the realignment of teeth, especially in canine teeth with rounded salience and large quantities of enamel in the buccal surface [2, 11, 12]. With regard to enamel removal, this aspect is significantly relevant, since wear can lead to tooth sensitivity, which occurs as a result of dentin exposure.

Selective enamel removal procedures have limitations, especially when compared with orthodontic treatment, since their indications are concerned. Orthodontic treatment can achieve the functional and esthetic requirements, minimizing and avoiding the wear of tooth [24]. On the other hand, if the patient is not inclined to receive this type of therapy and minor changes in the shape and position of the tooth are present, one of the alternative solutions to enhance the esthetic appearance of the smile is the enamel removal combined or not with direct restoration.

The patient's choice of treatment options is fundamental for the success of any procedure and it must be respected since it involves their psychological, physical, and financial aspects [25]. The patient in the present clinical case did not agree to have previous orthodontic therapy for tooth realignment. Therefore, tooth recontouring became a more feasible treatment option, taking into consideration the biological and functional concepts of dental structure preservation and esthetic demand. At the end of the procedure, the patient's satisfaction with the harmony obtained in her smile was achieved and undoubtedly functioned as an important instrument in the final evaluation of the result obtained.

In conclusion, the tooth recontouring techniques based on selective enamel removal and composite resin restorations provide a minimally invasive protocol with excellent esthetic result. This is an interesting restorative alternative based on a relatively simple treatment and lower-cost technique compared to traditional all ceramic restorations.

## References

[1] Okuda W. (2013). Developing a road map for success in esthetics. *General Dentistry*.

[2] Dietschi D. (2008). Optimizing smile composition and esthetics with resin composites and other conservative esthetic procedures. *European Journal of Esthetic Dentistry*.

[3] Claman L., Alfaro M. A., Mercado A. (2003). An interdisciplinary approach for improved esthetic results in the anterior maxilla. *Journal of Prosthetic Dentistry*.

[4] Kolawole K. A., Ayeni O. O., Osiatuma V. I. (2012). Psychosocial impact of dental aesthetics among university undergraduates. *International Orthodontics*.

[5] Olsen J. A., Inglehart M. R. (2011). Malocclusions and perceptions of attractiveness, intelligence, and personality, and behavioral intentions. *American Journal of Orthodontics and Dentofacial Orthopedics*.

[6] Tin-Oo M. M., Saddki N., Hassan N. (2011). Factors influencing patient satisfaction with dental appearance and treatments they desire to improve aesthetics. *BMC Oral Health*.

[7] Davis N. C. (2007). Smile design. *Dental Clinics of North America*.

[8] Bhuvaneswaran M. (2010). Principles of smile design. *Journal of Conservative Dentistry*.

[9] Shetty S., Pitti V., Satish Babu C., Surendra Kumar G., Jnanadev K. (2011). To evaluate the validity of recurring esthetic dental proportion in natural dentition. *Journal of Conservative Dentistry*.

[10] Calamia J. R., Levine J. B., Lipp M., Cisneros G., Wolff M. S. (2011). Smile design and treatment planning with the help of a comprehensive esthetic evaluation form. *Dental Clinics of North America*.

[11] Thordarson A., Zachrisson B. U., Mjör I. A. (1991). Remodeling of canines to the shape of lateral incisors by grinding: a long-term clinical and radiographic evaluation. *American Journal of Orthodontics and Dentofacial Orthopedics*.

[12] Zachrisson B. U., Mjör I. A. (1975). Remodeling of teeth by grinding. *American Journal of Orthodontics*.

[13] Wang Y., Sa Y., Liang S., Jiang T. (2013). Minimally invasive treatment for esthetic management of severe dental fluorosis: a case report. *Operative Dentistry*.

[14] Malhotra N., Mala K., Acharya S. (2011). Resin-based composite as a direct esthetic restorative material. *Compendium of Continuing Education in Dentistry*.

[15] Ardu S., Benbachir N., Stavridakis M., Dietschi D., Krejci I., Feilzer A. (2009). A combined chemo-mechanical approach for aesthetic management of superficial enamel defects. *British Dental Journal*.

[16] Edelhoff D., Sorensen J. A. (2002). Tooth structure removal associated with various preparation designs for anterior teeth. *Journal of Prosthetic Dentistry*.

[17] Nahsan F. P. S., Mondelli R. F. L., Franco E. B. (2012). Clinical strategies for esthetic excellence in anterior tooth restorations: understanding color and composite resin selection. *Journal of Applied Oral Science*.

[18] Akarslan Z., Sadik B., Erten H., Karabulut E. (2009). Dental esthetic satisfaction, received and desired dental treatments for improvement of esthetics. *Indian Journal of Dental Research*.

[19] Paranhos L. R., Lima C. S., da Silva R. H., Daruge Júnior E., Torres F. C. (2012). Correlation between maxillary central incisor crown morphology and mandibular dental arch form in normal occlusion subjects. *Brazilian Dental Journal*.

[20] Wolff D., Kraus T., Schach C. (2010). Recontouring teeth and closing diastemas with direct composite buildups: a clinical evaluation of survival and quality parameters. *Journal of Dentistry*.

[21] Gresnigt M., Ozcan M. (2011). Esthetic rehabilitation of anterior teeth with porcelain laminates and sectional veneers. *Journal of the Canadian Dental Association*.

[22] Greenberg J. R., Bogert M. C. (2010). A dental esthetic checklist for treatment planning in esthetic dentistry. *Compendium of Continuing Education in Dentistry*.

[23] Siéssere S., Vitti M., de Sousa L. G., Semprini M., Regalo S. C. H. (2004). Educational material of dental anatomy applied to study the morphology of permanent teeth. *Brazilian Dental Journal*.

[24] Bloom D. R., Padayachy J. N. (2006). Smile lifts—a functional and aesthetic perspective. *British Dental Journal*.

[25] Chu C. H., Zhang C. F., Jin L. J. (2011). Treating a maxillary midline diastema in adult patients—a general dentist's perspective. *Journal of the American Dental Association*.

